# Uroplakin Peptide-Specific Autoimmunity Initiates Interstitial Cystitis/Painful Bladder Syndrome in Mice

**DOI:** 10.1371/journal.pone.0072067

**Published:** 2013-08-16

**Authors:** Kenan Izgi, Cengiz Z. Altuntas, Fuat Bicer, Ahmet Ozer, Cagri Sakalar, Xiaoxia Li, Vincent K. Tuohy, Firouz Daneshgari

**Affiliations:** 1 Department of Urology, Case Western Reserve University, Cleveland, Ohio, United States of America; 2 Department of Clinical Chemistry, Cleveland State University, Cleveland,, Ohio, United States of America; 3 Department of Genetics, Case Western Reserve University, Cleveland, Ohio, United States of America; 4 Department of Immunology, Lerner Research Institute, Cleveland Clinic, Cleveland, Ohio, United States of America; University of Miami, United States of America

## Abstract

The pathophysiology of interstitial cystitis/painful bladder syndrome (IC/PBS) is enigmatic. Autoimmunity and impaired urothelium might lead the underlying pathology. A major shortcoming in IC/PBS research has been the lack of an appropriate animal model. In this study, we show that the bladder specific uroplakin 3A-derived immunogenic peptide UPK3A 65–84, which contains the binding motif for IA^d^ MHC class II molecules expressed in BALB/c mice, is capable of inducing experimental autoimmune cystitis in female mice of that strain. A highly antigen-specific recall proliferative response of lymph node cells to UPK3A 65–84 was observed, characterized by selectively activated CD4+ T cells with a proinflammatory Th1-like phenotype, including enhanced production of interferon γ and interleukin-2. T cell infiltration of the bladder and bladder-specific increased gene expression of inflammatory cytokines were observed. Either active immunization with UPK3A 65–84 or adoptive transfer of peptide-activated CD4+ T cells induced all of the predominant IC/PBS phenotypic characteristics, including increased micturition frequency, decreased urine output per micturition, and increased pelvic pain responses to stimulation with von Frey filaments. Our study demonstrates the creation of a more specific experimental autoimmune cystitis model that is the first inducible model for IC/PBS that manifests all of the major symptoms of this debilitating condition.

## Introduction

Interstitial cystitis (IC) is a chronic sterile inflammation of the bladder, inducing pain in the pelvic region and in the bladder [1]. The symptoms typically include a frequent and/or urgent need to urinate, and nocturia [2]. In order to include patients with IC symptoms in the absence of predominant bladder inflammation, the International Continence Society recently defined the broader term painful bladder syndrome (PBS) as “the complaint of suprapubic pain related to bladder filling, accompanied by other symptoms such as increased daytime and night-time frequency, in the absence of proven urinary infection or other obvious pathology” [3]. The most recent NIH-funded epidemiological study of IC/PBS in women in the U.S. (Rand IC Epidemiology or RICE Study) identified a prevalence of 6.5% and 2.7% based on high sensitivity and high specificity criteria, respectively, for diagnosing IC/PBS [4]. Those percentages translated into 3.3 to 7.9 million women 18 years old or older with IC/PBS symptoms [4]. Other studies have estimated the prevalence of IC/PBS among men to be 2 to 5 times lower than in women [5], [6]. Symptoms of IC interfere with employment, social relationships, and sexual activity. Furthermore, chronic pain, urinary frequency and urgency, and sleep deprivation associated with IC/PBS may contribute to psychological distress. Advancement in addressing this disease has been slow due to its uncertain etiology and a lack of understanding of the underlying pathophysiology.

Many possible pathophysiological mechanisms have been suggested for IC, including inflammatory, neurogenic, autoimmune, vascular or lymphatic disorders; damage to the glycosaminoglycan layer; and the occurrence of toxic substances in the urine [7]. It is possible that IC/PBS could have various etiologies, all of which result in parallel clinical manifestations. Support for an autoimmune etiopathogenesis has come from accumulating reports of associations of IC/PBS with autoimmune diseases such as lupus erythematosis, rheumatoid arthritis, ulcerative colitis, thyroiditis, Sjögren syndrome and fibromyalgia syndrome [8]–[11], as well as reports of higher occurrence of autoantibodies in the serum of IC patients [9]. In addition, antibodies that recognize uroepithelial cells were found in the urine of IC patients [10]. Higher numbers of CD4+, CD8+ and γδ T cells, as well as IgA, IgG and IgM plasma cells were found in the urothelium and submucosa of human IC bladder biopsies compared with normal bladder biopsies [12], [13]. IC also has been associated with the HLA DR6 allele of MHC class II as a relative risk factor [14].

One of the limitations in IC/PBS research has been the lack of an animal model that manifests all of the major symptoms, including both increased urinary frequency and chronic pelvic pain. Numerous animal models have been developed to help identify underlying mechanisms and possible treatment options for IC/PBS. These models include bladder inflammation induced by intravesical administration of an irritant or immune stimulant, virally induced neurogenic inflammation, chronic stress models, and experimental autoimmunity [15], [16].

The concept of experimental autoimmunity through the stimulation of pro-inflammatory type 1 T cell responses to targeted self-antigens has been utilized recently to create practical models of autoimmune conditions including autoimmune encephalomyelitis/multiple sclerosis [17], autoimmune myocarditis/dilated cardiomyopathy [18], autoimmune oophoritis/premature ovarian failure [19], autoimmune inner ear disease [20], and autoimmune cystitis [21]–[24]. We reported the creation of experimental autoimmune cystitis (EAC) models in mice that mimic the urinary phenotype of human IC [21], [22]. In the initial model, we induced bladder autoimmunity using a mouse bladder homogenate, which can also induce non-specific systemic autoimmune complications [21], while the second EAC model was generated by immunizing mice with recombinant uroplakin 2 [22]. We observed increased micturition frequency, but not chronic pain, in both of those models. Uroplakins (1A, 1B, 2, 3A, and 3B) are a family of heterodimeric integral membrane proteins that are part of the asymmetric unit membrane formed by terminally differentiated urothelial cells [25]. It has been suggested that uroplakins play a protective role by preventing the asymmetric unit membrane from breaching during bladder distension [26]. Expression of all of the uroplakins except 1B appears to be urothelium-specific, making them ideal targets for the establishment of EAC.

In the present study, we focused on creating an advanced and more specific EAC model by identifying a single peptide of uroplakin 3A that is recognized by IA^d^ MHC class II molecules found in BALB/c mice. We determined that a peptide consisting of residues 65–84 of murine uroplakin 3A (UPK3A 65–84), which contains an -SXXVXV- hexapeptide motif, was immunogenic in female BALB/c mice and capable of inducing a bladder-specific T cell-mediated response without any collateral or systemic autoimmune damage. Immunization with UPK3A 65–84 resulted in an autoimmune phenotype that included increased urinary frequency and chronic pelvic visceral pain, thus mimicking the clinical and histopathological characteristics of human IC/PBS. This study provides the only clinically relevant inducible murine model for IC/PBS that manifests all of the predominant symptoms of IC/PBS.

## Results

### UPK3A 65–84 Activates CD4+ Proinflammatory Th1-like T Cells in an IA^d^-restricted Manner

**Figure 1:**
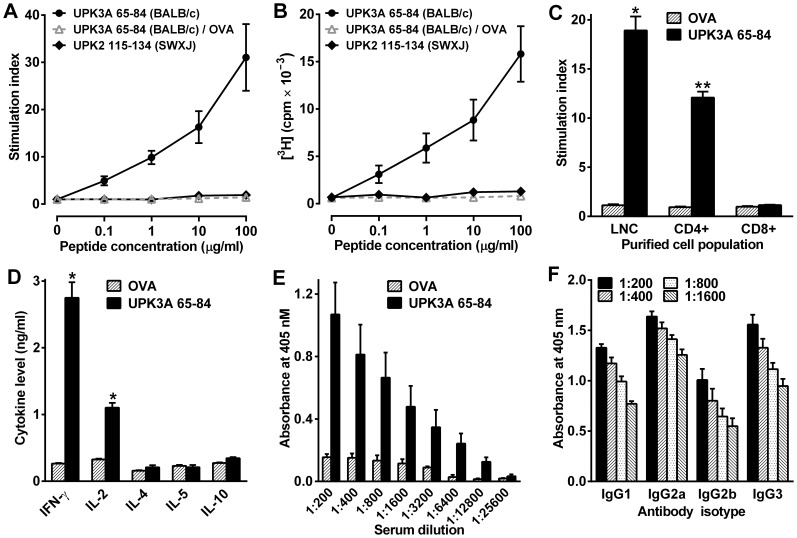
Characterization of the immune response to UPK3A 65–84. (**A–B**) Antigen specific recall proliferative responses of LNC taken from female BALB/c and SWXJ mice 10 days after immunization with UPK3A 65–84 and UPK2 115–134, respectively. Results of thymidine incorporation assays of BALB/c LNC incubated with serial dilutions of UPK3A 65–84 (filled circles) or OVA (open triangles), or SWXJ LNC incubated with serial dilutions of UPK2 115–134 (filled diamonds) are expressed as the mean plus and minus SEM of the stimulation index (A) or radioactivity (B) (n = 5 per group). (**C**) High antigen-specific recall responses to UPK3A 65–84 by LNC and purified CD4+ T cells, but not by CD8+ T cells, taken 10 days after immunization of female BALB/c mice with UPK3A 65–84 (10-day-primed LNC). Cells were cultured with peptide or OVA (10 µg/ml), and proliferation was measured by thymidine incorporation. Results are mean stimulation indices from 5 mice per group (*p = 0.0002 and **p = <0.0001 by unpaired t tests of UPK3A 65–84 vs. OVA pairs). (**D**) ELISA of cytokines in supernatants of 10-day-primed LNC cultured with UPK3A 65–84 or OVA for 48 h, demonstrating a proinflammatory type-1 response to UPK3A 65–84, with high production of IFNγ and IL-2 (n = 8 per group; *p<0.0001 by unpaired t tests of UPK3A 65–84 vs. OVA pairs). (**E–F**) Antibody responses of sera collected 5 weeks after immunization with UPK3A 65–84. (**E**) Total antibody titer. ELISA of sera (n = 5 per group) with anti-mouse IgG antibody showed high titer antibody responses to UPK3A 65–84, but not to OVA. (**F**) Isotype-specific antibody titer. ELISA of sera (n = 4 per group) with IgG isotype-specific antibodies revealed a predominantly type-1 antibody response to UPK3A 65–84 involving high production of IgG2a and IgG3 compared with IgG1 and IgG2b. Two way ANOVA with Tukey’s multiple comparisons tests of the different IgG isotypes in (F) revealed that titers of IgG2a differed significantly from those of IgG1 and IgG2b at all dilutions (*p*<0.01), while titers of IgG3 differed significantly from those of IgG2b (*p*<0.01), but not IgG1, at all dilutions. All assays were performed in triplicate for each mouse; error bars in C–F indicate plus SEM.

The 20-mer UPK3A 65–84 peptide derived from the mouse bladder specific protein uroplakin 3A was predicted by the SYFPEITHI program and database to be highly immunogenic in BALB/c mice, due to its inclusion of the -SXXVXV- binding motif for IA^d^ MHC class II molecules expressed in those mice. Injection of this peptide into female BALB/c mice yielded high cellular and humoral immunogenicity, shown by recall proliferation assays of lymph node cells (LNC) and antibody responses of sera taken from immunized mice (Figure 1). LNC obtained from mice 10 days after immunization with UPK3A 65–84 (10-day-primed LNC) proliferated in response to UPK3A 65–84, but were unresponsive to ovalbumin (OVA) as a control (Figure 1A,B). The magnitude of the peptide-specific recall response to UPK3A 65–84 was similar to the response to recombinant uroplakin 2 that we reported previously [22]. On the other hand, LNC obtained from female SWXJ mice immunized with peptide UPK2 115–134 exhibited a negligible recall response to the UPK2 peptide (Figure 1A,B), suggesting either that other peptide sequences of uroplakin 2 were responsible for its immunogenicity in our previous study, or additional native UPK2 amino acid residues are required in order for the –KXXS- motif to be properly processed and presented by MHC.

CD4+ T cells purified (>90%) from UPK3A 65-84-immunized BALB/c 10-day-primed LNC proliferated in response to UPK3A 65–84, whereas purified CD8+ T cells were unresponsive (Figure 1C). Accordingly, UPK3A 65–84 preferentially actuates CD4+T cells restricted to the IA^d^ haplotype of MHC class II molecules. ELISA analysis of 48 hour supernatants of 10-day-primed LNC cultured with UPK3A 65–84 or OVA revealed a proinflammatory Th1-like cytokine recall response to UPK3A 65–84 characterized by enhanced expression of IFNγ and IL-2, and virtually no production of Th2-associated IL-4, IL-5, or IL-10 (Figure 1D). Total and isotype-specific antibody titers were determined in sera collected from mice 5 weeks after immunization with UPK3A 65–84. A high titer (1∶12,800) systemic serum antibody response was observed to UPK3A 65–84, but not to OVA (Figure 1E). In 2 of 5 mice, immunoreactivity was detectable at a dilution of 1∶25,600. The predominant antibody isotypes produced in response to the peptide were IgG2a and IgG3, which are known to be induced by IFN-γ and inhibited by IL-4 [27], [28] (Figure 1F). That finding, along with somewhat lower production of Th2-associated IgG1 and TGFβ-induced IgG2b, indicated that the antibody response to the UPK3A 65–84 peptide is predominantly a Th1-associated response.

### Bladder Specific Inflammatory Response in EAC Mice

**Figure 2:**
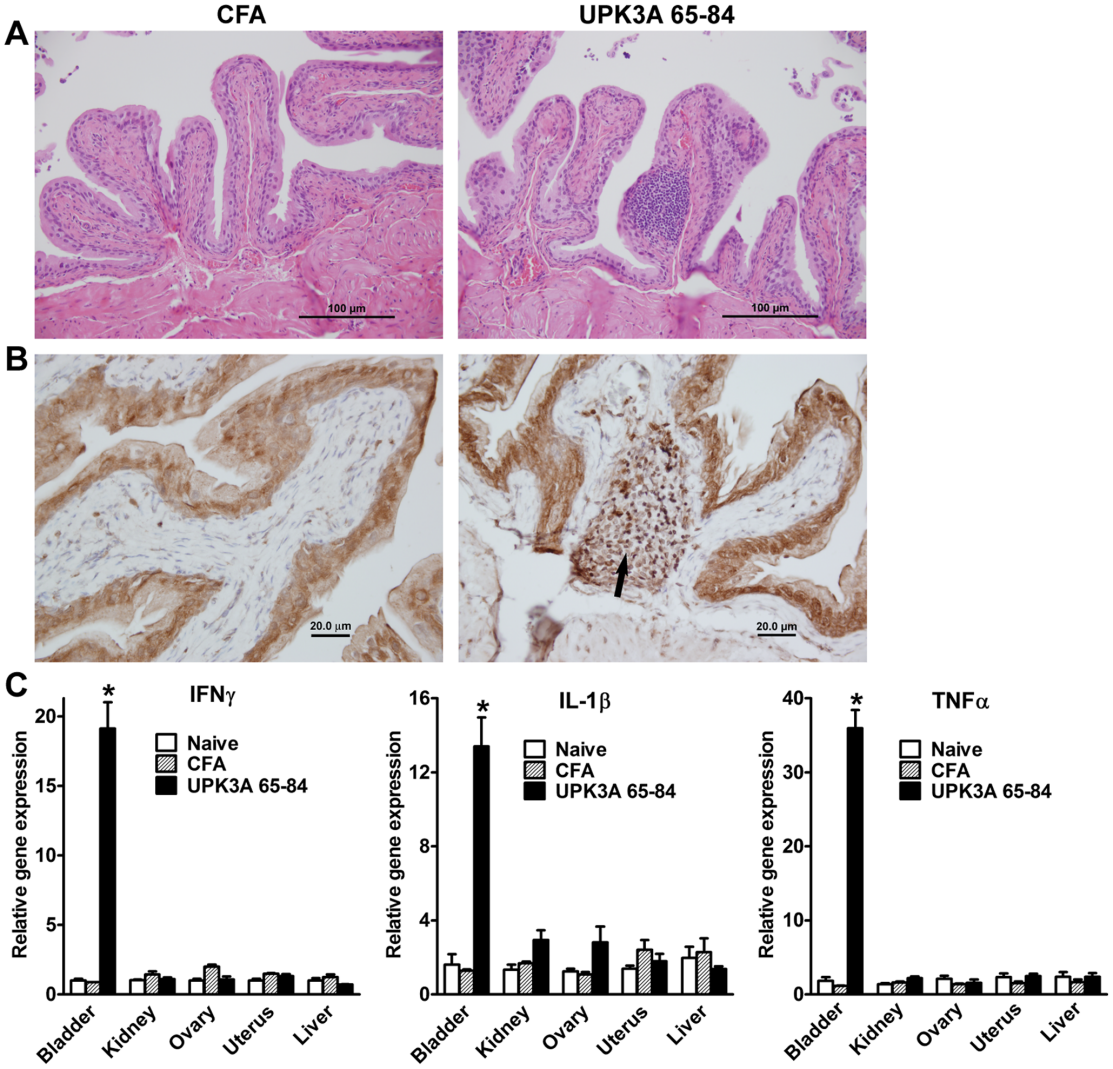
Bladder-specific inflammation in mice 5 weeks after immunization with UPK3A 65–84. (**A**) Hematoxylin and eosin stained bladder sections taken from UPK3A 65-84-immunized mice showed extensive perivascular infiltration (right panel) not evident in sections from CFA*-*immunized control mice (left panel). Solid bar = 100 µm. (**B**) Immunostaining with CD3 antibody showed a predominance of T cells in bladder infiltrates of mice immunized with UPK3A 65–84 (right panel), but no evidence of T cell infiltration in bladder sections from CFA-immunized control mice (left panel). Solid bar = 20 µm. (**C**) qRT-PCR analysis showed significantly elevated levels of IFNγ, IL-1β, and TNFα in the bladder but not in the kidney, ovary, uterus, or liver of mice immunized with UPK3A 65–84 compared to tissues from age- and sex-matched naïve mice or control mice immunized with CFA. Error bars indicate plus SEM of 4 mice per group, with triplicate assays for each mouse. *p<0.0001 for UPK3A 65-84-immunized mice vs. CFA-immunized or naïve mice by one way ANOVA with Tukey’s multiple comparisons test.

Hematoxylin and eosin stained bladder sections taken 5 weeks after immunization with UPK3A 65–84 showed extensive perivascular infiltration of inflammatory response cells (Figure 2A, right) that was not evident in any sections taken from control mice immunized with complete Freund’s adjuvant (CFA) (Figure 2A, left). Immunohistochemical staining for T cell determinant CD3 showed that the bladder infiltrating cells were predominantly T cells (Figure 2B, right), and that no such T cell infiltration was evident in bladder tissues from control mice (Figure 2B, left). The bladder T cell accumulation in UPK3A 65-84-immunized mice was predominantly located under the urothelium, in the area of lamina propria. qRT-PCR analysis showed significantly elevated gene expression levels of the inflammatory cytokines IFNγ, IL-1β, and TNFα in the bladder of UPK3A 65-84-immunized mice, but not in the kidney, ovary, uterus, or liver of those mice, and not in any of those tissues taken from age- and sex-matched naïve or CFA-immunized control mice (Figure 2C). Thus, we have demonstrated that the inflammation in EAC mice is confined to bladder tissue.

### IC/PBS Phenotype in EAC Mice

**Figure 3:**
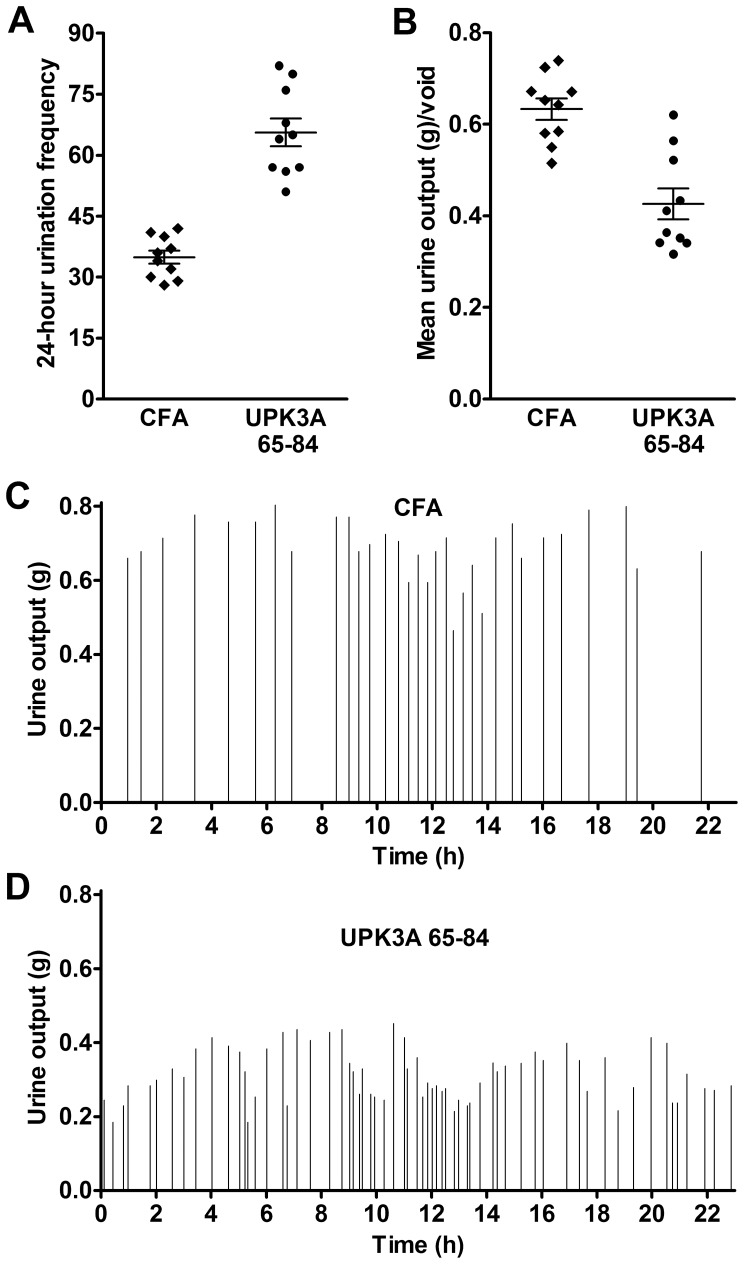
Bladder dysfunction in mice immunized with UPK3A 65–84. (**A**) 24-hour micturition frequencies were significantly higher 5 weeks after immunization of female BALB/c mice with UPK3A 65–84 compared to control mice immunized with CFA (*p*<0.0001). (**B**) Inversely, mean urine output/micturition was significantly lower in mice immunized with UPK3A 65–84 compared with control mice immunized with CFA (*p* = 0.0001). Error bars indicate plus and minus SEM. (**C–D**) The graphs show the urine production and frequency of a representative individual mouse immunized with UPK3A 65–84 (C) and one immunized with CFA (D).

**Figure 4:**
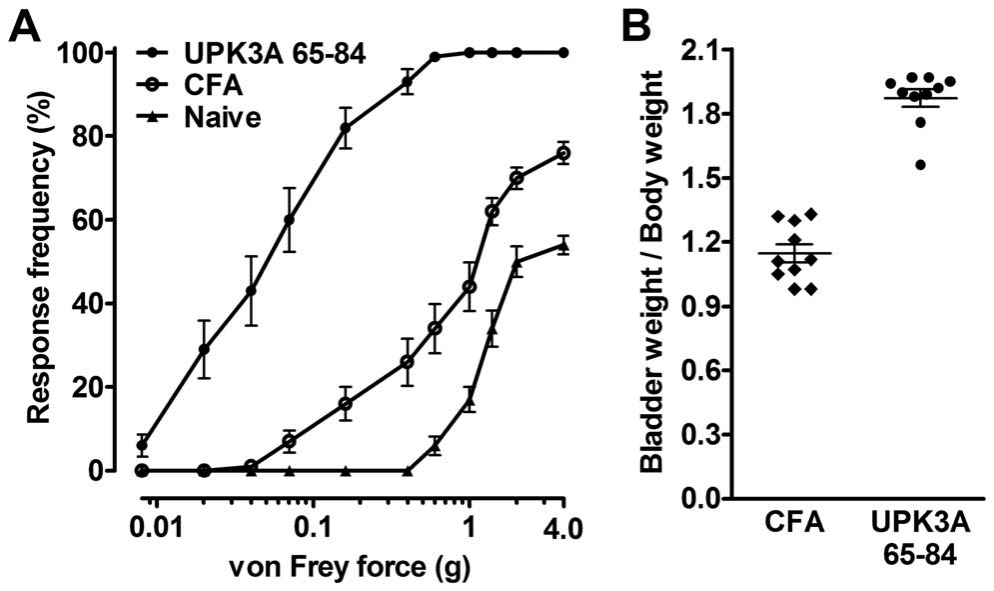
Pelvic hyperalgesia and increased bladder weight in mice immunized with UPK3A 65–84. (**A**) Pain responses were assessed by application of von Frey filaments to the suprapubic pelvic region in mice 5 weeks after immunization with UPK3A 65–84 or CFA, or in naïve mice (n = 10 per group). The response frequencies (percentage of positive responses out of 10 stimuli) to increasing filament forces were plotted, and 50% threshold forces were calculated as described in Methods. Mice immunized with UPK3A 65–84 exhibited significantly higher sensitivity (lower 50% thresholds, 0.059±0.046 g) in responding to suprapubic application of von Frey filaments, as an indicator of pain referred from the bladder, compared with CFA-immunized mice and naïve mice (1.1±0.56 g and 2.9±0.78 g, respectively; *p*<0.0001 in both comparisons by one way ANOVA of log-transformed 50% thresholds with Tukey’s multiple comparisons test). In the same analysis, the 50% thresholds of CFA-immunized and naïve mice also differed significantly from each other (*p*<0.01)**.** (**B**) Ratios of bladder weight (mg) to body weight (g) were significantly higher in mice immunized with UPK3A 65–84 compared to CFA-immunized controls (*p*<0.0001 by unpaired t test). Error bars indicate plus and minus SEM.

To determine if UPK3A 65–84 was able to induce the major phenotypes of IC/PBS, bladder dysfunction by urinary frequency-volume chart (FVC) and pelvic pain were measured in female BALB/c mice 5 weeks after immunization. Mice immunized with UPK3A 65–84 showed significantly increased micturition frequencies (Figure 3A) and significantly decreased mean urine outputs per micturition (Figure 3B) compared with control mice immunized with CFA. Figure 3 parts C and D show the FVC results of individual mice immunized with UPK3A 65–84 and CFA, respectively. This result mimics the phenotypical features of urinary frequency and urgency seen in human IC/PBS. Suprapubic pelvic pain in the mice was assessed using the widely-accepted, non-invasive von Frey monofilaments described in other studies of visceral pain [29], [30]. Mice immunized with UPK3A 65–84 exhibited significantly lower thresholds of response to tactile stimuli, as an indicator of referred pain from the bladder, compared with CFA-immunized and naïve mice (Figure 4A). The much smaller, though significant effect on 50% thresholds by CFA immunization compared with naïve mice suggests an inflammatory response to CFA that is presumably systemic. Moreover, UPK3A 65-84-immunized mice had significantly increased bladder weight to body weight ratios compared with CFA-immunized mice, a characteristic of animal models of IC indicative of bladder remodeling, possibly due to inflammation (Figure 4B). We thus confirm that active immunization with UPK3A 65–84 peptide is capable of inducing autoimmune cystitis in female BALB/c mice.

### IC/PBS Phenotype Induced by Adoptive Transfer of UPK3A 65-84-activated CD4+ T Cells

**Figure 5:**
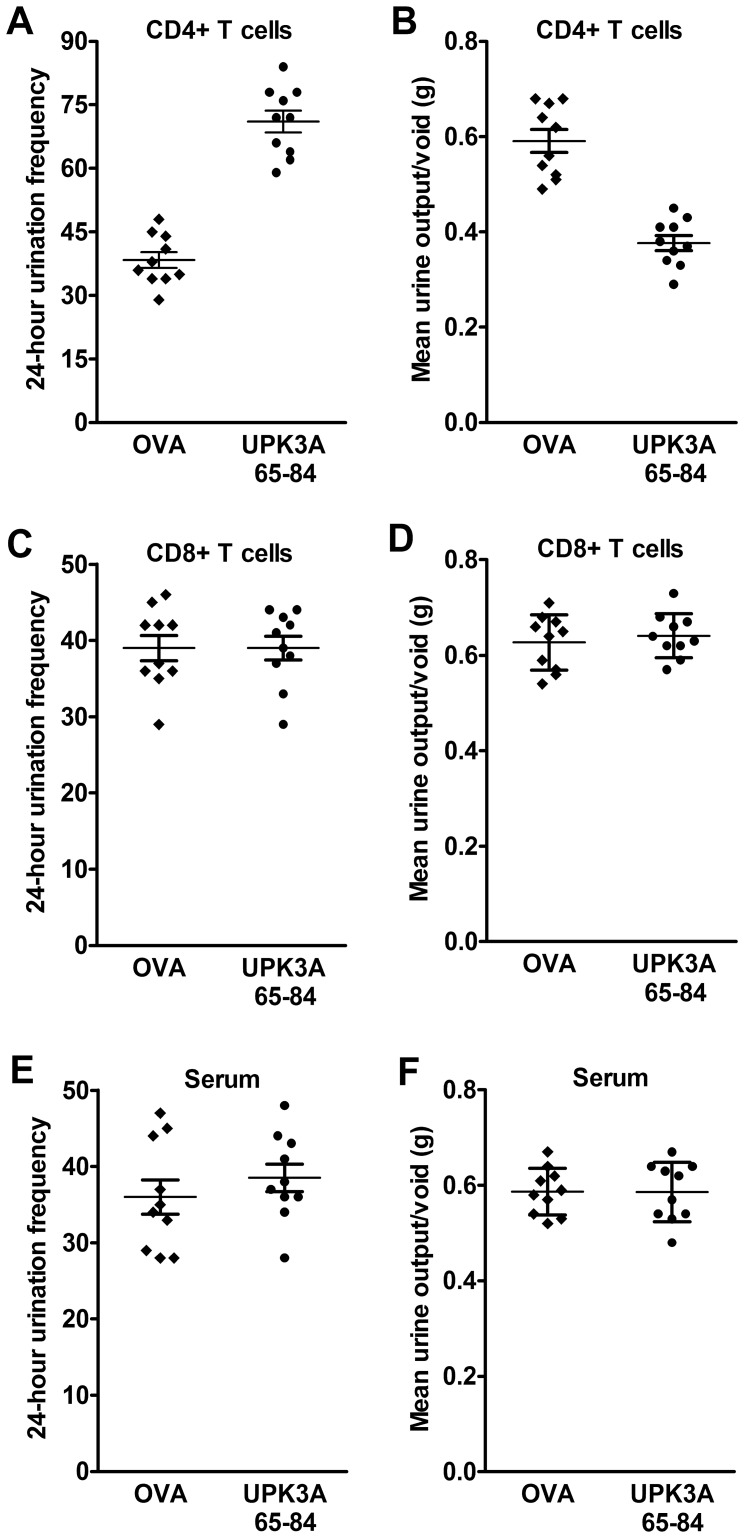
Adoptive transfer of EAC micturition phenotype. (**A–D**) Ten days after immunization of mice with UPK3A 65–84 peptide or OVA, CD4+ and CD8+ T cells were isolated, activated with immunogen, and transferred into naïve female BALB/c recipients by i.v. injection. Twenty four hour micturition (FVC) was measured 20 days after adoptive transfer (n = 10). Micturition frequency was significantly higher in mice injected with UPK3A 65-84-primed and -activated CD4+ T cells (**A**) (*p*<0.0001), but not CD8+ T cells (**C**), compared to control OVA-induced T cells. Inversely, urine output per micturition was significantly lower in mice with UPK3A 65-84-induced CD4+ T cells (**B**) (*p*<0.0001), but not CD8+ T cells (**D**), compared to OVA-induced T cells. (**E,F**) Serum was collected 5 weeks after immunization of mice with UPK3A 65–84 or OVA, transferred into naïve female BALB/c recipients, and FVC was measured 20 days later, revealing no significant differences in micturition frequency (**E**) or urine output per micturition (**F**) between groups (n = 10). Error bars indicate plus and minus SEM.

**Figure 6:**
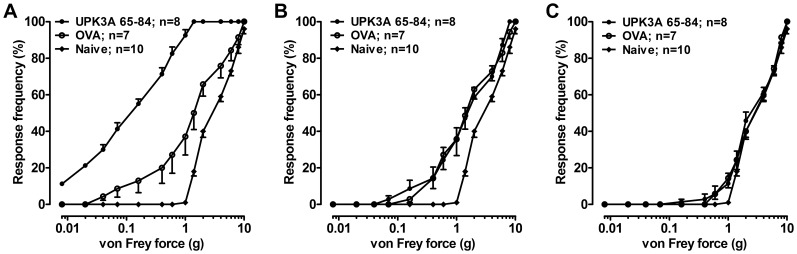
Adoptive transfer of EAC pelvic pain. Pelvic pain was assessed by application of von Frey filaments to the suprapubic pelvic region of female BALB/c mice 10 days after adoptive transfer of 10-day immunogen-primed and -activated CD4+ or CD8+ T cells, or serum. (**A**) UPK3A 65-84-induced CD4+ T cells yielded increased responses to stimulation compared with CD4+ T cells from OVA-immunized and uninjected naïve mice (*p*<0.01 and *p*<0.0001, respectively, by one way ANOVA of log-transformed 50% thresholds with Tukey’s multiple comparison test). In the same analysis, the 50% thresholds of OVA-immunized and naïve mice also differed significantly from each other (*p*<0.01) (**B**) UPK3A 65-84-induced CD8+ T cells yielded similar responses to stimulation of the suprapubic region compared with OVA-induced CD8+ T cells, though both were more sensitive to stimulation than uninjected naïve mice (*p*<0.01 in each case)**.** (**C**) Serum samples collected 5 weeks after immunization of mice with UPK3A 65–84 or OVA were transferred into naïve female BALB/c recipients by 3 i.v. injections of 200 µl serum per mouse. Application of von Frey filaments 10 days later revealed no significant differences in responses among uninjected naïve mice and recipients of serum from UPK3A 65–84- or OVA immunized mice. Error bars indicate plus and minus SEM.

We determined if the immunologic and functional features of EAC could be adoptively transferred to naïve mice by CD4+ T cells, CD8+ T cells, or serum isolated from directly immunized mice. Ten days after immunization with UPK3A 65–84 peptide or OVA, CD4+ and CD8+ T cells were isolated, activated with immunogen, and transferred into naïve female BALB/c recipients. Twenty days after adoptive transfer, the IC/PBS phenotype of bladder dysfunction was evident in mice injected with UPK3A 65-84-induced CD4+ T cells compared to control OVA-induced CD4+ T cells, as shown by increased micturition frequency and decreased urine output per micturition (Figure 5A,B). However, no signs of bladder dysfunction by FVC measurements were found in naïve recipients of UPK3A 65-84-induced CD8+ T cells compared to OVA-induced CD8+ T cells (Figure 5C,D). Similarly, sera collected 5 weeks after immunization of mice with UPK3A 65–84 or OVA and then transferred to naïve mice yielded no differences in urination between the groups 20 days after transfer (Figure 5E,F). Pelvic pain, the most prominent phenotype of IC/PBS, was examined 10 days after adoptive transfer, by applying von Frey filaments to the suprapubic pelvic region. Transfer of 10-day UPK3A 65-84-primed and -activated CD4+ T cells into naïve mice yielded significantly increased pain responses 10 days later compared with control mice that received OVA-induced CD4+ T cells specific for OVA**,** suggesting a lower threshold of pain referred from the bladder organ (Figure 6A)**.** On the other hand, adoptive transfer of 10-day UPK3A 65-84-induced CD8+ T cells into naïve recipients yielded no difference in pain assessment compared to transfer of control OVA-induced CD8+ T cells, although both of those induced small, but significant increases in pain responses compared with uninjected naïve mice (Figure 6B), as did OVA-induced CD4+ T cells (Figure 6A). Presumably, the CD4+ and CD8+ T cells induced by the known T cell immunogenicity of OVA caused a minor non-specific systemic inflammatory response when injected into naïve mice, as did the CD8+ T cells from UPK3A 65-84-immunized mice, which did not respond to UPK3A 65–84 in the proliferation assays (Figure 1C). On the other hand, sera collected 5 weeks after immunization of mice with UPK3A 65–84 or OVA and then injected into naïve mice had no effects on pain measurements compared to uninjected naïve mice (Figure 6C). Thus, serum from UPK3A 65-84-immunized mice, despite typically having a high titer of antibodies against UPK3A 65–84 (Figure 1E), was unable to induce an inflammatory response in the absence of a cellular response. In conclusion, the essence of these experiments is that passive transfer of CD4+ T cells, but not CD8+ T cells or serum, isolated from UPK3A 65–84-immunized mice induced EAC in naïve BALB/c mice with the characteristic phenotypical features of IC/PBS, namely frequent urination, decreased urine output per micturition, and increased pelvic pain responses, similar to primary immunization with UPK3A 65–84.

## Discussion

Our study reveals that a UPK3A peptide can induce a peptide-specific CD4+ T cell autoimmunity that mediates painful bladder dysfunction in mice. This model exhibits the phenotypical features of increased urinary frequency and pelvic pain seen in human IC/PBS. Advancement in research in IC/PBS has been overwhelmingly slow due to a lack of understanding of the underlying pathophysiology and a lack of reliable markers or animal models for the disease. The current approaches in our scientific area focus mostly on merging clinical practice and translational research, thus stressing the importance of translational models.

Almost 20 animal models that partly resemble the IC/PBS phenotype have been introduced during the past two decades [31], [32]. The earlier models depend on an inflammatory bladder insult, such as intravesical instillation of a chemical irritant [e.g., acetone [33], acid [34], acrolein [35], turpentine, mustard oil, or croton oil [36]], an immune response factor [antimicrobial peptide LL-37 (human cathelicidin) [37]], or bacterial lipopolysaccharide [38]; systemic administration of cyclophosphamide [39], [40]; pseudorabies virus infection of CNS bladder circuits [41], [42]; or systemic induction of bladder-directed autoimmunity (URO-OVA, URO-OVA/OT-I, bladder homogenate, uroplakin 2 protein) [17], [21], [22], [30], [43]–[45]. The intravesically applied compounds damage the glycosaminoglycan layer and bladder mucosa nonselectively, involving a variety of mechanisms that may not be relevant to human IC/PBS [32]. Cyclophosphamide-induced cystitis is a convenient and well-studied model, however, its pathogenesis results from direct toxicity to the bladder mucosa by its metabolite acrolein, which is excreted in the urine [40]. The magnitude of the increased suprapubic pain responses and micturition defects we observed in our EAC model appear to be at least as high as were observed in cyclophosphamide-induced cystitis in male and female C57BL/6 mice [46]. Injection of pseudorabies virus into the abductor caudae dorsalis tail muscle of a rat induces neurogenic cystitis without directly affecting the bladder, but bladders typically become acontractile within 5 days of virus injection, thus the model does not accurately recapitulate all aspects of human IC/PBS [47]. Transgenic URO-OVA mice produce a membrane form of OVA as a self-antigen on the urothelium and are susceptible to bladder inflammation induced by adoptive transfer of activated OVA-specific T cells [43]. URO-OVA/OT-I mice, derived by crossing URO-OVA mice with transgenic mice that express an OVA-specific CD8+ T cell receptor (OT-I mice), spontaneously develop autoimmune cystitis [43]. However, OVA is not an endogenous antigen of bladder, and the bladder urodynamic changes and pain correlates of the IC/PBS phenotype have not been characterized in those transgenic models [43], [44]. A spontaneous model of IC/PBS was described in cats [48], however this has not been studied extensively in the scientific community due its unknown etiology and the high costs of maintaining and handling cats, which require involvement of a veterinarian [15].

In our earlier reports, we employed bladder homogenate [21] and the bladder specific protein uroplakin 2 [22] for immunization of mice, and assessed the effects on bladder function. The disadvantage of immunization with homogenate is that its composition includes bladder specific and non-specific antigens, thus can induce non-specific and systemic immune reactions in addition to bladder-targeted reactions. Our model of uroplakin 2-immunized mice provided bladder specific autoimmunity, but those mice did not demonstrate enhanced pelvic pain responses to noxious stimuli, thus missing one of the major symptoms of IC/PBS. Mice immunized with bladder homogenate or uroplakin 2 did exhibit increased urination frequencies and decreased urine output per void [21], [22]. The similarity of those observed abnormalities with the urinary symptoms of human IC/PBS encouraged us to develop a more potent, targeted autoimmunity against a bladder-specific antigen and determine if it would induce the pelvic pain phenotype together with increased frequency and decreased volume per void, the cardinal manifestations of IC/PBS.

In our present study, we evaluated the immunogenicity of peptides identified in bladder specific uroplakin proteins. We found that a 20-mer peptide from uroplakin 3A, UPK3A 65–84, is highly immunogenic in female BALB/c mice and specifically induces a CD4+ T cell-mediated immune response. The immunized mice showed significantly elevated urinary frequencies, decreased urine outputs per void and increased pelvic pain responses, reproducing all three major symptoms of human IC/PBS. Correspondingly, the passive transfer of UPK3A 65-84-restricted CD4+ T cells into naïve BALB/c recipients induced the same EAC phenotypes as direct immunization.

Our data indicate that the bladder dysfunction occurring in UPK3A 65-84-induced EAC is due to T cell-mediated autoimmune inflammation of the bladder tissue. We verified inflammation in bladder by revealing increased expression of proinflammatory cytokines and the presence of clusters of T cells in the bladder following immunization. Moreover, the significantly increased bladder weight to body weight ratios of EAC mice are indicative of organ remodeling, likely due to inflammation in this case. The finding of bladder specific inflammation that directs persistent bladder dysfunction parallels the defects observed in uroplakin 2- or 3-null mice [49].

In this study, we evaluated referred hyperalgesia of the bladder using von Frey filaments on the suprapubic pelvic region [50]. We found early enhanced responses to normally painful stimuli and painful responses to normally painless stimuli after adoptive transfer of UPK3A 65-84-induced CD+4 T cells. These results inspire additional investigations of the mechanism of pelvic pain in this model. The role of mast cells in the induction of cystitis pain has been reported [51], [52]. Our EAC model is suitable for studies of the involvement of mast cells and their regulation by T cells in the development of IC/PBS-related pelvic pain.

With regard to the reported association of IC/PBS with the HLA DR6 allele of MHC class II [14], we found it interesting that the extracellular domains of human uroplakins 1A and 2, though not uroplakin 3A, contain potential HLA-DR6 binding sites, according to the published HLA-DR6 binding motifs [53]. Those sequences (human UPK1A 171–179, sequence **F**TS**A**F**R**AA**T**, and human UPK2 117–125, sequence **I**SY**L**V**K**KG**T**) suggest that uroplakins 1A and 2 are possible targets for T cell-mediated autoimmunity leading to IC/PBS in humans.

The significance of our model is that a single peptide, UPK3A 65–84, induces T cell-dependent autoimmune-mediated EAC with high bladder specificity that is unique in accurately reflecting both the urinary symptoms and chronic pelvic pain of IC/PBS. Along with a near 100% rate of induction of EAC, these findings qualify this animal model as a realistic, potentially useful model for future exploration of the pathogenesis and therapeutic intervention of IC/PBS. Such a translational model of IC/PBS is greatly needed and will be valuable for accelerating efforts to understand and treat this chronic, debilitating disease in humans.

## Materials and Methods

### Ethics Statement

All mouse protocols were pre-approved by the Institutional Animal Care and Use Committee of Case Western Reserve University (IACUC permit #2009-0131) in compliance with the Public Health Service policy on humane care and use of laboratory animals. All dissections were performed with the mice under isoflurane anesthesia, and were followed by euthanasia with an overdose of sodium pentobarbital. All efforts were made to minimize suffering.

### Peptide Identification and Synthesis

We employed an online database of MHC molecules and their recognized peptide motifs (http://www.syfpeithi.de/) [54] to locate potentially immunogenic peptides for BALB/c mice (haplotype H-2^d^) in the sequences of the bladder-specific uroplakin proteins. A peptide consisting of residues 65–84 of uroplakin 3A, containing the -SXXVXV- binding motif for IA^d^ MHC class II molecules (UPK3A 65–84, sequence AMVDSAM**S**RN**V**S**V**QDSAGVP), was predicted by the SYFPEITHI program and database to be highly immunogenic in BALB/c mice. We also identified a potentially immunogenic peptide for SWXJ mice (hybrid haplotype H-2^q,s^) in the sequence of uroplakin 2, the protein that yielded the urinary phenotype of IC/PBS when used to immunize female SWXJ mice [22]. The peptide UPK2 115–134 from the extracellular domain of uroplakin 2, sequence YYISYRVQ**K** GT**S**TESSPETP, contains the reported -KXXS- binding motif for IA^s^ and IA^q^ MHC class II molecules [18]. The 20-mer UPK3A 65–84 and UPK2 115–134 peptides were synthesized by the Molecular Biotechnology Core Facility of the Lerner Research Institute. The peptides were purified by reverse-phase HPLC and their amino acid compositions were confirmed by mass spectrometry.

### Mice and Immunization

Female BALB/c mice were purchased from Jackson Laboratory. At 6–8 weeks of age, mice were injected s.c. in the abdominal flank with or without 200 µg of UPK3A 65–84 peptide or ovalbumin (OVA; Sigma-Aldrich, St. Louis, MO) in 200 µl of an emulsion of equal volumes of water and Freund’s adjuvant containing 400 µg of *Mycobacterium tuberculosis* H37RA (complete Freund’s adjuvant [CFA], Difco Laboratories, Detroit, MI), or with the emulsion of water and CFA alone.

### Cell Culture and Proliferation Assays

To assess the immunogenicity of the UPK3A 65–84 peptide [18], [19], inguinal and axillary LNCs were removed from mice 10 days after immunization (10-day-primed LNC) and cultured for thymidine incorporation assays. The cells were plated at 3×10^5^ cells/well in a single-cell suspension in 96-well flat-bottom microtiter plates (Falcon, BD Biosciences, San Jose, CA) with Dulbecco modified Eagle medium (DMEM) (Mediatech CellGro, Herndon, VA) containing 10% fetal bovine serum (HyClone), 5% HEPES buffer, 2% L-glutamine, and 1% penicillin/streptomycin (Invitrogen Life Technologies). Serial 10-fold dilutions of peptide or OVA (negative control), or 2 µg/ml of anti-mouse CD3 (BD Biosciences; positive control) were added to triplicate wells and the cells were incubated at 37°C for 96 hours in humidified air with 5% CO_2_. The cells were then pulsed with [methyl-^3^H] thymidine (l µCi per well; specific activity 6.7 Ci/mmol; New England Nuclear, Boston, MA), and sixteen hours later the cells were harvested by aspiration onto glass fiber filters. The level of incorporated radioactivity was determined by scintillation spectrometry. The results are shown as mean counts per minute (cpm) of LNC cultures from 5 mice and as the mean ratio of cpm in cultures with antigen to cpm in cultures without antigen [stimulation index; [18]].

### T Cell Isolation

The proliferative responses of CD4+ and CD8+ T cells to the UPK3A 65–84 peptide were determined. CD4+ and CD8+ T cells were purified from 10-day UPK3A 65-84-primed LNC by positive selection with anti-CD4- and anti-CD8-coated magnetic beads, respectively, using a MACS LS column in a Midi MACS cell separator (Miltenyi Biotec, Auburn, CA, USA). The purified (>90%) CD4+ or CD8+ T cells were plated in 96-well Falcon microtiter plates at 3×10^5^ cells/well with 5×10^5^ cells/well gamma-irradiated (2000 rads) syngeneic splenocyte feeders, and 50 µg/ml of UPK3A 65–84 peptide or OVA (negative control), or 2 µg/ml of anti-mouse CD3 (BD Biosciences; positive control) was added into triplicate cultured wells. The cells were incubated at 37°C for 96 hours and then proliferation was measured by [methyl-^3^H] thymidine incorporation as described above.

### Cytokine Analysis

The concentrations of cytokines IFN-γ, IL-2, IL-4, IL-5, and IL-10 were determined by ELISA in culture medium of 10-day-primed LNC cultured in the presence of 25 µg/ml UPK3A 65–84 or OVA (negative control) for 48 hours. Purified capture/detection antibody pairs and recombinant cytokines were obtained commercially (BD Biosciences) and included anti-mouse IFN-γ (R4-6A2 and biotin XMG1.2), anti-mouse IL-2 (JES6-1A12 and biotin JES6-5H4), anti-mouse IL-4 (11B11 and biotin BVD6-24G2), anti-mouse IL-5 (TRFK5 and biotin TRFK4), and anti-mouse IL-10 (JES5-2A5 and biotin SXC-1). ELISA was performed as described in our previous studies [19], [22], and absorbance was measured at 405 nm using a Versamax ELISA microplate reader (Molecular Devices, Sunnyvale, CA). Standard curves were established using known concentrations of each recombinant cytokine, and sample cytokine concentrations were determined from values within the linear part of the standard curve.

### Total Antibody Titer

Five weeks after immunization of mice with UPK3A 65–84 (n = 5), the total antibody titer in serum was determined by ELISA. Serial dilutions of serum samples were incubated in microtiter plate wells coated with UPK3A 65–84 antigen or OVA. Goat anti-mouse IgG antibody (H+L chain specific) conjugated to HRP (Southern Biotech) was then used at 1/8000 for detection, and absorbance was measured at 405 nm.

### Isotype-Specific Antibody Titers

Isotype-specific antibody titers to UPK3A 65–84 were determined in serum samples obtained from mice 5 weeks after immunization with UPK3A 65–84 (n = 4). Microtiter wells coated with UPK3A 65–84 antigen were blocked with BSA and incubated with serial dilutions of the serum samples (1/200, 1/400, 1/800, and 1/1600). Using a mouse MonoAB ID/SP ELISA kit (Zymed-Invitrogen, Carlsbad, CA) according to the manufacturer’s instructions, biotinylated antibodies specific to each mouse IgG isotype were added, followed by detection with streptavidin-HRP and 2,2′-azino bis (3-ethylbenzothiazoline-6-sulfonic acid) substrate, and measurement of absorbance at 405 nm.

### Histologic Analysis

Mice were euthanized 5 weeks after immunization with UPK3A 65–84 or CFA alone, and bladders were removed and weighed. The bladder was sectioned at the equatorial midline, fixed in 10% neutral formalin, dehydrated, and embedded in paraffin. Serial 5-µm tissue sections were placed on microscope slides, dewaxed, and rehydrated for routine hematoxylin and eosin staining. Gross histologic observations were performed using light microscopy (Olympus DP70 digital microscope).

### Immunocytochemistry

CD3 immunostaining of bladder tissues was performed as described [19]. Briefly, unmasked and blocked, formalin-fixed, paraffin-embedded 5-µm tissue sections were treated with a 1∶250 dilution of rat anti-mouse CD3 antibody (Novacastra, Newcastle Upon Tyne, UK) followed by a 1∶100 dilution of mouse-adsorbed biotinylated goat anti-rat IgG (BD Biosciences). Slides were developed conventionally using streptavidin-HRP (Vectastain^®^ ABC kit, Vector Laboratories, Burlingame, CA) with 3,3′-diaminobenzidine chromogen and hydrogen peroxide substrate solution (BioGenex, San Ramon, CA) and examined by light microscopy (Olympus DP70 digital microscope).

### Real Time quantitative Reverse Transcription - Polymerase Chain Reaction (qRT-PCR)

Expression levels of mRNAs for inflammatory proteins IFN-γ, IL-1β, and TNF-α were measured in tissues of mice 5 weeks after immunization with UPK3A 65–84 or CFA alone, and in naïve mice tissues. Mice were euthanized and total RNA was extracted from bladder, kidney, ovary, uterus, and liver using TRIzol^®^ reagent (Invitrogen, Carlsbad, CA). cDNA was synthesized from RNA using Super Script III cDNA synthesis kit with random hexamer primers (Invitrogen). The primer pairs for qRT-PCR were designed using the online Universal Probe Library Assay Design Center (Roche, Mannheim, Germany). The sequences of the primers used are: IFN γ, TGATGGCCTGATTGTCTTTCAA (sense) and GGATATCTGGAGGAACTGGCAA (antisense); IL-1β, GAGTGTGGATCCCAAGCAAT (sense) and AGACAGGCTTGTGCTCTGCT (antisense); TNF α, CAAAGGGAGAGTGGTCAGGT (sense) and ATTGCACCTCAGGGAAGAGT (antisense); β-actin, GGTCATCACTATTGGCAACG (sense) and ACGGATGTCAACGTCACACT (antisense). qRT-PCR was performed using a SYBR Green PCR Master kit with an ABI Prism 7500 Sequence Detection System (Applied Biosystems, Foster City, CA). Cytokine gene expression levels normalized to expression of housekeeping gene β-actin and relative to the average level in naïve mice for each tissue were calculated with the comparative C_T_ method [55], after confirming that the mean levels of β-actin mRNA did not differ significantly between the EAC and CFA mice.

### Urinary Frequency-volume Chart (FVC) Assessment

Twenty four hours prior to FVC assessment, solid food was eliminated from cages and replaced with lactose-free milk to minimize the frequency and mass of feces produced during the time of examination [56]. Micturition and drinking behaviors of mice 5 weeks after immunization were measured continuously over 24 hours by placing each mouse individually in a metabolic cage (MED-CYT-M; Med-Associates, St. Albans, VT) and collecting urine in a plastic tray located on an analytical balance (VI- 3 mg; Acculab, Huntingdon Valley, PA) set directly underneath the cage. Balances were linked to a data acquisition software program advanced by the manufacturer, which record and total the weight of urine collected over the defined period of time. Throughout testing, mice were provided with free access to lactose-free milk and water, and the testing room was maintained on the usual light/dark cycle. The amounts of milk and water in the provided bottles were measured at the beginning and end of testing to quantify fluid consumption over the 24-hour period.

### Pain Assessment

Pelvic visceral pain responses of mice were assessed 5 weeks after immunization using calibrated von Frey monofilaments [57]. A set of 12 von Frey filaments (Stoelting Co., Wood Dale, IL) of sizes 1.65 to 5.07 was used, corresponding to an approximately logarithmic scale of forces (0.008, 0.02, 0.04, 0.07, 0.16, 0.4, 0.6, 1.0, 1.4, 2.0, 4.0, 6.0, 8.0 and 10.0g), and providing a linear scale of perceived intensity. Tactile sensitivity of the suprapubic pelvic region was assessed by applying von Frey filaments perpendicularly to the surface and recording the responses of the mice. The behaviors that were considered to be a positive response were: 1) sharp withdrawal of the abdomen, 2) instant licking and scratching, or 3) jumping. Beginning with the smallest filament, each filament was applied a total of 10 times for 3 seconds, with intervals of 8 seconds between each stimulus. The results are expressed as the percentage of positive responses for each monofilament (response frequency). In addition, the von Frey force defined as that which would elicit a response 50% of the time (50% threshold) was calculated for each mouse from the regression line drawn through the linear portion of a plot of response frequency vs. log of the von Frey force, using GraphPad Prism 5 (GraphPad Software, Inc., La Jolla, CA).

### Adoptive Transfer of EAC

To determine whether the immunologic and functional features of EAC could be transferred from primary-immunized mice to naïve mice, CD4+ T cells and CD8+ T cells were isolated from female BALB/c mice that had been immunized 10 days earlier (at 6–8 weeks of age) with 200 µg of UPK3A 65–84 or OVA. Lymph node cells were harvested and activated with the corresponding immunogen (50 µg/ml) in vitro for 96 hours, and then CD4+ and CD8+ T cells were isolated by magnetic bead separation as described above. The purified T cells were injected i.v. into the tail vein of naïve female BALB/c mice (2×10^7^ cells in 200 µl PBS per mouse). In addition, serum samples were collected from mice 5 weeks after immunization with UPK3A 65–84 or OVA, and sera were injected i.v. into naïve mice (3 injections of 200 µl spaced 48 h apart per mouse). The EAC phenotype was assessed by measuring micturition frequency and volume, and pelvic pain via von Frey filaments 20 and 10 days after adoptive transfer, respectively.

### Statistical Analysis

The unpaired, two-tailed Student’s t-test was used to analyze differences between experimental and control groups in stimulation index (Fig. 1C), LNC production of cytokines, micturition frequency, mean urine output per void, and bladder weight/body weight ratio, using Welch’s correction in cases where the variances were significantly different. One way ANOVA with Tukey’s multiple comparisons test was used to compare cytokine gene expression and pain perception (log-transformed 50% thresholds) among three groups. Isotype-specific antibody titers were analyzed by two way ANOVA with Tukey’s multiple comparisons test to compare different IgG isotypes at each dilution. Values of *p*<0.05 were considered statistically significant.
